# A Case of Medication-Resistant Acanthamoeba Keratitis Treated by Corneal Crosslinking in Turkey

**DOI:** 10.1155/2013/608253

**Published:** 2013-12-22

**Authors:** Goktug Demirci, Akif Ozdamar

**Affiliations:** ^1^Department of Ophthalmology, Medipol University Hospital, 34800 Istanbul, Turkey; ^2^Department of Ophthalmology, Cerrahpasa Medical Faculty of Istanbul University, 34800 Istanbul, Turkey

## Abstract

*Purpose*. To report a case of medication-resistant acanthamoeba keratitis (AK) treated successfully by corneal crosslinking (CXL). *Methods*. A 26-year-old male with medication-resistant AK underwent a standard CXL procedure with local anesthesia, followed by central corneal epithelial debridement, application of riboflavin 0.1%, and UV-A irradiation. *Results*. The patient experienced a dramatic symptomatic improvement within 24 hours. At two months, keratitis was healed with a semitransparent paracentral scar that did not affect visual acuity. *Conclusions*. Our experience, considered in the context of recent studies, suggests that CXL may be an option for selected patients with medication-resistant AK and corneal melting. CXL allows patients to avoid emergency keratoplasty and experience rapid symptomatic relief.

## 1. Introduction

Acanthamoeba keratitis (AK) is a rare, vision-threatening, necrotizing corneal disease caused by small, free-living amoebae belonging to the genus Acanthamoeba. They are typically uninucleated, active, motile trophozoites but form a double-walled cyst and become dormant when exposed to harsh environmental conditions [1]. The first AK case was reported in 1974 and case reports increased in 1984, particularly among contact lens wearers [2].

AK typically presents with severe pain and associated radial neuritis, inflammation, redness, and photophobia. AK is the most clinically challenging form of ophthalmologic keratitis. In the past, AK symptoms often led ophthalmologists to incorrectly diagnose herpetic keratitis. Mathers et al. showed that, in some series of herpetic keratitis, AK was also present as an opportunistic infection [3]. Combination drug therapy is highly effective only with early diagnosis. With late diagnosis, medical therapy may be insufficient to stop disease progression, and penetrating keratoplasty (PKP) may be required [4].

Japanese scientists demonstrated that, when riboflavin is exposed to visible light or UV light, it can inactivate RNA-containing tobacco mosaic virus [5]. Recently, several studies have demonstrated the efficacy of UV light in inactivating medication-resistant AK [6, 7]. Considering this successful application, we performed CXL for the treatment of medication-resistant AK.

## 2. Case

**Figure 1:**
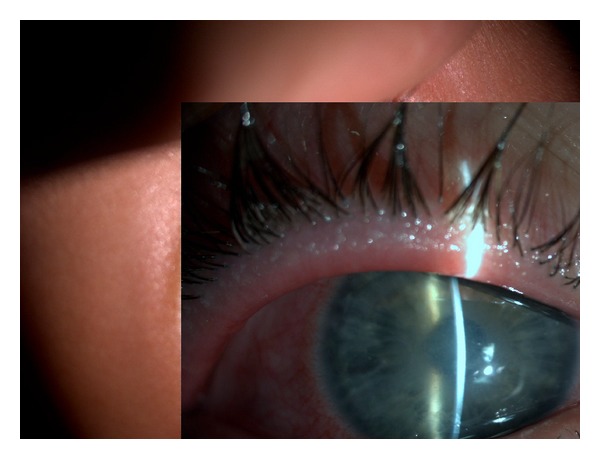
Ring ulcer and stromal necrosis of cornea.

**Figure 2:**
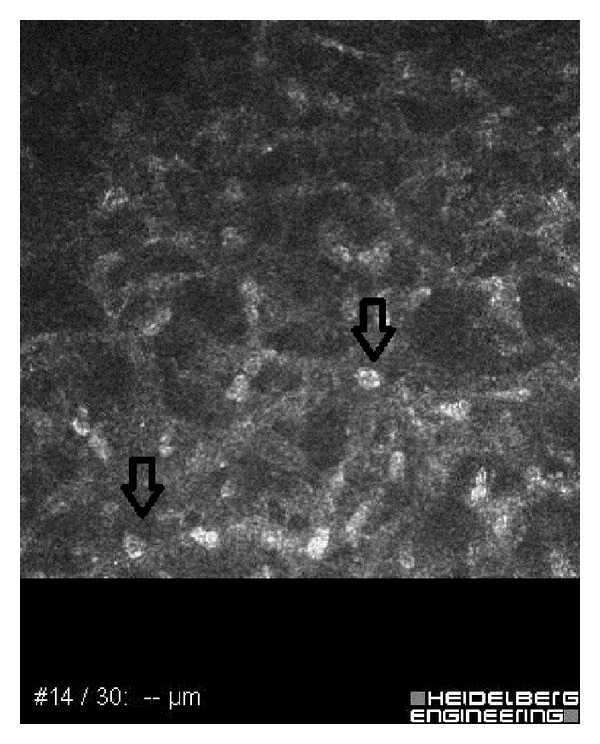
Confocal corneal examination revealing double-walled acanthamoebal cysts.

**Figure 3:**
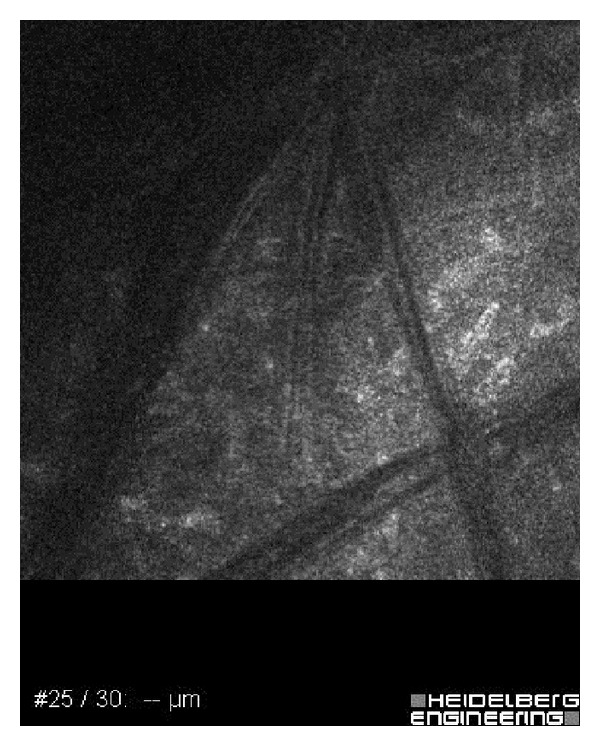
Confocal corneal examination revealing extreme corneal nerve thickening.

A 26-year-old male with a history of soft contact lens use was referred to our ophthalmology clinic with severe pain, redness, and photophobia in the right eye. Two months ago, he had received topical antibiotics from a general ophthalmologist. Because the corneal ulcer did not heal, he was referred to a corneal specialist. Confocal microscopy and corneal scraping cultures demonstrated acanthamoeba infection. Despite antiacanthamoebal treatment with 1 mg/mL propamidine isethionate eye drops (Brolene, Aventis Pharma) hourly and chlorhexidine 0.02% eye drops (200 mu g/mL) hourly, the patient's keratitis worsened. Physical examination revealed a ring ulcer, typical of acanthamoeba, with stromal necrosis (Figure 1). Confocal corneal examination revealed double-walled acanthamoebal cysts throughout the cornea (Figure 2), as well as extreme corneal nerve thickening (Figure 3). Based on our assessment of medication-resistant acanthamoeba keratitis with melting, we performed CXL according to the Dresden protocol and forewent immediate PKP.

**Figure 4:**
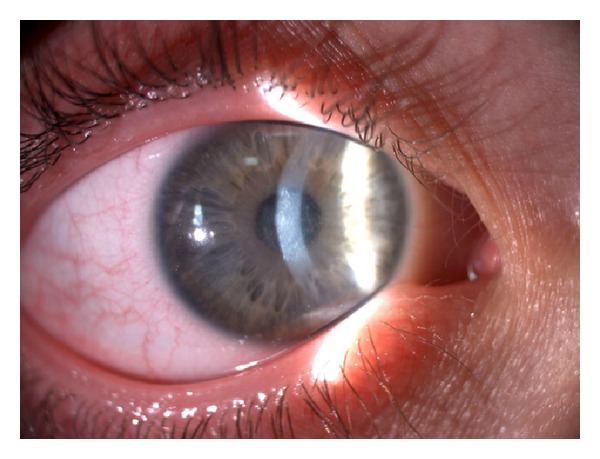
Biomicroscopy of cornea at the end of first month revealing complete resolution of keratitis.

CXL treatment for AK keratitis is nearly identical to standard protocol keratoconus treatment. However, they differ in that only loose epithelium and epithelium around the infectious site are removed following anesthetic eye drop application in AK keratitis. Following administration of topical 0.1% tetracaine every 5 minutes for 15 minutes, we debrided the central 9 mm of corneal epithelium and acanthamoebal infiltrates. Next, 0.1% aqueous riboflavin phosphate (vitamin B2) drops were administered every 3 min for 30 min. The eye was then irradiated for 30 minutes with UV-A, at a working distance of 5 cm. Riboflavin was reapplied every 5 minutes during the 30-minute irradiation period. The patient experienced dramatic symptomatic improvement, especially pain reduction, over 24 hours. Moxifloxacin drops (Vigamox, Alcon) were administered four times daily. All ancillary inflammatory signs resolved after several days. Corneal melting was arrested and complete epithelialization was achieved in 10 days. During the first month of follow-up, there were no signs of active keratitis by clinical exam or confocal microscopy. At one month, we prescribed dexamethasone 0.1% eye drops (Dexa-Sine SE, Liba) (Figure 4). After two months, keratitis healed, and the patient had a semitransparent paracentral scar that did not affect visual acuity.

## 3. Discussion

Most patients presenting with AK are soft contact lens wearers [3]. It is thought that contact lenses create corneal abrasions by microtrauma, which facilitates acanthamoebal entry. Animal studies show that amoebae are pathologic in the context of compromised epithelium [8, 9]. Tap water rinsing of contact lenses allows deposition of lime scale deposits, which often contain pathogenic *Acanthamoeba* species [10]. However, some AK patients do not have this history, and persons who do not wear contact lenses have also been affected by AK [11].

Combination drug therapy is highly successful when the disease is diagnosed early, but there is no gold-standard treatment. In contrast to bacterial ulcers, AKs do not progress rapidly; therefore, the practitioner has an opportunity to successfully intervene, even if signs and symptoms have been present for one month [9].

In 2008, Iseli et al. described a series of five patients who underwent successful CXL to treat infectious corneal melts which had failed conventional antimicrobial pharmacotherapy. They suggested two possible mechanisms for immediate regression of the corneal melting process [12]. First, CXL may increase collagen resistance to digestive enzymes such as pepsin, trypsin, and collagenase. Approximately 90% of corneal thickness is stroma, which is composed of regularly arranged collagen fibrils with keratocytes. Bacteria and fungi produce enzymes that digest human collagen and cause corneal melting. Secondly, cell apoptosis following CXL may occur for both human keratocytes and pathogens due to the antimicrobial effect of UV-A light [12, 13]. Several case reports have documented responses to CXL therapy in complicated infectious keratitis [6, 7, 14, 15]. In 2010, Makdoumi et al. performed the first prospective study of CXL as a first-line treatment for bacterial corneal ulcers. Of 16 patients receiving CXL as primary therapy for suspected bacterial keratitis, only two required antibiotic treatment [14]. The antibacterial effectiveness of CXL has been demonstrated *in vitro* against *Staphylococcus aureus*, *Staphylococcus epidermidis,* and *Streptococcus pneumoniae* grown on agar plates. Martins et al. showed limited effects for *Pseudomonas aeruginosa* and no measurable effect for *Candida albicans* [16]. *In vitro* effects of UV-A and riboflavin on acanthamoebae are less impressive than those observed *in vivo* effects. However, *in vitro* results do not always translate to *in vivo* efficacy [17, 18].

Despite these promising results, the above-referenced studies were limited by small case numbers. Further investigations are needed before CXL can be incorporated into routine clinical practice for keratitis treatment.

Our experience of a rapid response with a rapid subjective reduction in pain and photophobia differs from experiences with CXL for keratoconus, in which pain remains until epithelization completion. This difference may be due to the rapid atrophic keratoneuritis response of corneal nerve plexus; it was reported that subepithelial stromal nerve fibers disappeared immediately after CXL and were restored one year later with full corneal sensitivity [19]. Makdoumi et al. have also reported rapid symptomatic relief and believe that this results from ocular immune-response effects, which may be involved in corneal melting through stromal immune-cell modulation [14]. Khan et al. theorize that oxidative processes during CXL may deplete the natural nutritional source to microorganisms, thus acting as a cysticidal agent. In addition, activated flavin and free radical insult to DNA or RNA may damage microorganism genomes. Others believe that the direct damaging effect of UV light is unlikely to produce antimicrobial effects [7].

In consideration of our current understanding of CXL, this rapid response may be due to thermomechanical harm to AK trophozoites, which would be exposed to temperatures of nearly 75°C in the anterior stroma during this procedure [20].

## 4. Conclusion

To our knowledge, this is the first reported case of successful CXL treatment for AK keratitis in Turkey. Our case suggests that CXL may be a promising option in selected cases of medication-resistant AK with corneal melting. CXL treatment may allow patients to avoid emergency keratoplasty and provide rapid symptomatic relief, but further investigations are needed.
